# P34 Targeting antimicrobial prescribing knowledge gaps among Foundation doctors through a focused microbiology education programme

**DOI:** 10.1093/jacamr/dlag102.040

**Published:** 2026-06-26

**Authors:** Mecaelan Sardar, Darshana Wickramasinghe

**Affiliations:** Medway NHS Foundation Trust, Kent, UK; Medway NHS Foundation Trust, Kent, UK

## Abstract

**Background:**

Antimicrobial stewardship relies on junior doctors making safe, early prescribing decisions. At a district general hospital in the UK, repeated microbiology enquiries from Foundation doctors suggested baseline knowledge deficits. Identifying local learning needs can help inform focused microbiology education and promote safer prescribing.

**Objectives:**

To identify key antimicrobial prescribing knowledge gaps and use these findings to inform targeted microbiology education aligned with local Trust guidelines.

**Methods:**

Cycle 1 involved an anonymized baseline quiz completed by 22 Foundation doctors during a scheduled teaching session. Domains assessed included antimicrobial spectrum of activity, first-line treatment for *Clostridioides difficile* infection, criteria for treating asymptomatic bacteriuria (ASB), principles of candidaemia management, and infection prevention knowledge. Findings informed a multifaceted educational intervention comprising: (1) immediate quiz debrief, (2) a 2-h consultant microbiologist-led teaching session, (3) bedside teaching reinforcement during microbiology ward rounds, and (4) signposting to local antimicrobial guidelines.

**Results:**

Five key knowledge gaps were identified. Only 50% (11/22) selected the correct guideline-recommended first-line therapy for initial *C. difficile* infection, and 45.5% (10/22) chose an incorrect dose or duration. Recognition of the urgency of candidaemia was low, with just 4.5% (1/22) acknowledging that *Candida* in blood cultures is never a contaminant. ASB management showed a consistent tendency toward overtreatment of scenarios where antibiotics are not indicated: for example, 59.1% (13/22) would treat ASB before orthopaedic surgery. Spectrum-of-activity misunderstanding was notable: 68.2% (15/22) incorrectly believed that gentamicin provided anaerobic coverage, and recognition of antipseudomonal agents was frequently suboptimal. In contrast, 86.4% (19/22) correctly identified that ‘before touching patient surroundings’ is not one of the WHO Five Moments for Hand Hygiene.

**Conclusions:**

A simple baseline assessment successfully identified specific, modifiable knowledge gaps in antimicrobial stewardship among Foundation doctors. Targeted microbiology teaching was developed to address these areas. A re-audit is planned to evaluate improvements in knowledge and their impact on safer prescribing practice.

